# Instance of Perforation of the Duodenum Occasioning Death after Twenty Hours Illness

**Published:** 1846-10

**Authors:** George Stillwell

**Affiliations:** Surgeon, Epsom


*Instance of Perforation of the Duodenum occasioning death after twenty hours illness.* By George Stillwell, Esq., Surgeon, Epsom.—A gentleman who had previously enjoyed excellent and uninterrupted health, had, for three days, slight uneasiness in the stomach and bowels, which induced him to take a dose of castor-oil, but did not prevent his entering fully into his occupations and amusements. On his return from the Downs, where he had passed several hours on horseback, on Friday, the 29th of May; he was seized, at a short distance from Epsom, at 6 o'clock, P. M. with violent pains in the stomach and bowels, which obliged him to alight, and seek a place to lie down. He entered a farm-house, and threw himself on his abdomen, apparently in great agony, and seeking relief from pressure. A considerable quantity of brandy and hot water was administered, which appeared to afford some slight abatement of pain. When I first visited the patient, about half an hour after his entering the farm-house, he was lying on his back on a sofa, in a state of collapse, complaining of severe pain in the region of the stomach; his countenance livid; extremities cold, and pulse not more than thirty beats per minute. Having had him removed, and placed in bed, warmth was applied in various modes externally, and warm diluents were plentifully administered; the natural heat was by these means restored, and the pulse rose to sixty. Medicines were now given to procure evacuation from the bowels, and were continued with injections, both simple and medicated, but very slight fæcal discharge was produced by the use of these remedies, and no real relief afforded. During the night, and up to ten o'clock on Saturday morning, although the symptoms were extremely urgent, and the paroxysms of pain of frequent recurrence, still they were not such as to preclude all hope of permanent relief. The pulse had continued at 60; the countenance and general bearing of the patient evinced strength and fortitude, and his conversation was not exclusively devoted to his illness. At 12 o'clock, an interval of two hours having elapsed since my last attendance, I found my patient again in a state of collapse, in which he quickly sank, remaining perfectly collected until within a few minutes of breathing his last. There was only one attempt at vomiting during the progress of the disease.

The rapidly fatal course of the disease suggested its nature, and the cause of death; which was confirmed by the post mortem examination of the body twenty hours afterwards, conducted with the assistance of another medical gentleman, who had concurred in the treatment of the case.

The external appearances were discoloration and tumefaction. An unusual quantity of offensive gas escaped on opening the cavity of the abdomen, which contained a large quantity of fluid mixed with the various liquids, &c. which had been taken into the stomach. These had passed through an ulcer larger than a fourpenny piece, which had perforated the duodenum on its anterior surface, near its junction to the pylorus. The peritoneal surfaces evinced marks of inflammatory action, with some effusion of coagulable lymph; the stomach was in a healthy state, with the exception of slight emphysema of the internal coat, the result of incipient putrefaction. From the thickening of the edges of the ulcer, and the want of morbid vascularity, the disease most probably had been gradual in its progress, and of lengthened duration.--*Ib.*

